# Use of a risk scoring tool to identify higher-risk HIV-1 serodiscordant couples for an antiretroviral-based HIV-1 prevention intervention

**DOI:** 10.1186/s12879-016-1899-y

**Published:** 2016-10-17

**Authors:** Elizabeth M. Irungu, Renee Heffron, Nelly Mugo, Kenneth Ngure, Elly Katabira, Nulu Bulya, Elizabeth Bukusi, Josephine Odoyo, Stephen Asiimwe, Edna Tindimwebwa, Connie Celum, Jared M. Baeten, for the Partners Demonstration Project Team, Jared Baeten, Connie Celum, Renee Heffron, Deborah Donnell, Ruanne Barnabas, Jessica Haberer, Harald Haugen, Craig Hendrix, Lara Kidoguchi, Mark Marzinke, Susan Morrison, Jennifer Morton, Norma Ware, Monique Wyatt, Elly Katabira, Nulu Bulya, Elizabeth Bukusi, Josephine Odoyo, Nelly Mugo, Kenneth Ngure

**Affiliations:** 1 College of Health Sciences, Jomo Kenyatta University of Agriculture and Technology, P. O. Box 62000-00200, Nairobi, Kenya; 2 Department of Global Health, University of Washington, 325 Ninth Avenue, Seattle, WA 98104 USA; 3 Department of Epidemiology, University of Washington, 325 Ninth Avenue, Seattle, WA 98104 USA; 4 Department of Medicine, University of Washington, 325 Ninth Avenue, Seattle, WA 98104 USA; 5 Centre for Clinical Research, Kenya Medical Research Institute, Mbagathi Road, Nairobi, Kenya; 6 Department of Medicine, Makerere University, PO Box 7072, Kampala, Uganda; 7 Infectious Disease Institute, College of Health Sciences, Makerere University , Kampala, Uganda; 8 Department of Obstetrics & Gynaecology, University of Nairobi, Uhuru Highway, Nairobi, 00100 Kenya; 9 Center for Microbiology Research, Kenya Medical Research Institute, Mbagathi Road, Nairobi, Kenya; 10 Kabwohe Clinical Research Centre, Off Kabwohe-Ishaka HWY next to ICOBI, Kabwohe-Itendero Town Council, Box 347, Bushenyi District Kabwohe, Uganda

**Keywords:** HIV, Serodiscordant couples, Screening tool, PrEP, Delivery, Africa

## Abstract

**Background:**

Antiretroviral therapy (ART) and pre-exposure prophylaxis (PrEP) reduce HIV-1 transmission within heterosexual HIV-1 serodiscordant couples. Prioritizing couples at highest HIV-1 transmission risk for ART and PrEP would maximize impact and minimize costs.

**Methods:**

The Partners Demonstration Project is an open-label, delivery study of integrated PrEP and ART for HIV-1 prevention among high risk HIV-1 serodiscordant couples in Kenya and Uganda. We evaluated the feasibility of using a validated risk score that weighs a combination of easily measurable factors (age, children, marital status, male circumcision status, condom use, plasma HIV-1 levels) to identify couples at highest risk for HIV-1 transmission for enrollment. Couples scoring ≥5 met the risk score eligibility criteria.

**Results:**

We screened 1694 HIV-1 serodiscordant couples and enrolled 1013. Of the screened couples, 1331 (78.6 %) scored ≥5 (with an expected incidence >3 % per year) and 76 % of these entered the study. The median age of the HIV-1 uninfected partner was 29 years [IQR 26, 36] and 20 % were <25 years of age. The HIV-1 uninfected partner was male in 67 % of partnerships, 33 % of whom were uncircumcised, 57 % of couples had no children, and 65 % reported unprotected sex in the month prior to enrollment. Among HIV-1 infected partners, 41 % had plasma viral load >50,000 copies/ml.

**Conclusion:**

A risk scoring tool identified HIV-1 serodiscordant couples for a demonstration project of PrEP and ART with high HIV-1 risk. The tool may be feasible for research and public health settings to maximize efficiency and minimize HIV-1 prevention costs.

## Background

In sub-Saharan Africa, heterosexual HIV-1 serodiscordant couples account for a substantial proportion of new HIV-1 infections [1–3]. Randomized clinical trials have provided definitive evidence that antiretroviral treatment (ART) for HIV-1 infected persons [4] and pre-exposure prophylaxis (PrEP) for HIV-1 uninfected persons are highly efficacious in decreasing HIV-1 transmission risk within HIV-1 serodiscordant partnerships [5]. Mathematical modeling studies have found that providing HIV-1 serodiscordant couples with antiretroviral prevention interventions may have a significant impact on the HIV-1 epidemic [6]. Delivery of antiretroviral based HIV-1 prevention interventions, particularly in resource-constrained settings, must target those at highest risk for HIV-1, which would achieve maximal public health benefit in a cost-effective manner [7–9].

**Fig. 1:**
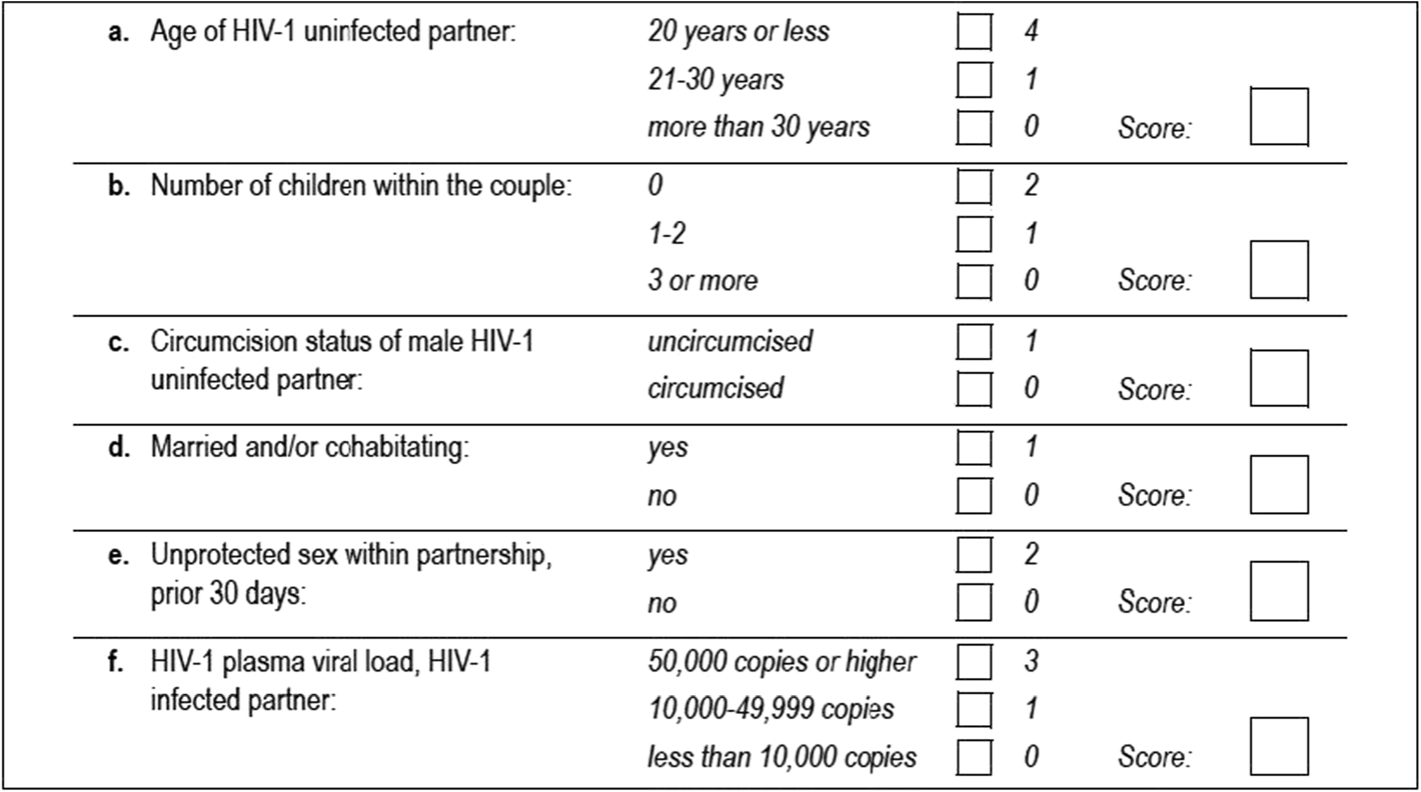
HIV-1 risk score worksheet

We previously developed and validated an empiric risk scoring tool to identify highest-risk HIV-1 serodiscordant African heterosexual couples using data from 8500 stable HIV-1 serodiscordant African couples enrolled in three prospective studies [10]. The score is composed of variables that are easily measurable in clinical settings including whether the couple had any unprotected sex in the prior month, the number of children in the partnership, marital status, age of the HIV-1 uninfected partner, circumcision status of HIV-1 uninfected male partners, and plasma HIV-1 RNA concentrations in the HIV-1 infected partner (Fig. 1). Additional variables such as gender, duration of partnership, hormonal contraception use and CD4 count were considered for the scoring tool but these were less predictive of HIV-1 transmission risk than the included factors. The maximum score is 12 and a score of 0–2 has an anticipated HIV-1 incidence of <1 % per year, 3–4 has an anticipated incidence of approximately 2 % per year, and a score ≥5 has an anticipated HIV-1 incidence of >3 % per year [10]. In the present analysis, we assessed the ability of the HIV-1 risk score to identify higher-risk HIV-1 serodiscordant couples for recruitment into a prospective study delivering ART and PrEP for HIV-1 prevention in Kenya and Uganda.

## Methods

### Study design

The Partners Demonstration Project is a prospective, open-label cohort study of the delivery of antiretroviral-based HIV-1 prevention to high-risk HIV-1 serodiscordant couples in four sites in Kenya and Uganda. HIV-1 infected partners are offered ART according to the national ART initiation guidelines which, as of 2014, recommend ART for all HIV-1 infected partners in serodiscordant couples, regardless of CD4 count [11]. PrEP is offered to the HIV-1 uninfected partner prior to and for the first 6 months after ART initiation by the infected partner, at which time viral suppression is expected.

### Recruitment and participant selection

Between November 2012 and August 2014, HIV-1 serodiscordant couples were recruited for the Partners Demonstration Project from HIV-1 voluntary counseling and testing (VCT) centers, HIV-1 care providers, programs for prevention of mother-to-child HIV-1 transmission (PMTCT) services, and community promotion activities for couples’ VCT. Enrollment was completed in August 2014. At the screening visit, demographic and behavioral information were collected via interviewer-administered standardized questionnaires. Screening laboratory tests included repeat HIV-1 testing for both partners following the national HIV-1 testing algorithms, serum creatinine and hepatitis B surface antigen (HBsAg) for HIV-1 uninfected partners, and CD4 count and plasma HIV-1 RNA concentrations for HIV-1 infected partners.

Based on data collected during screening, we computed the HIV-1 risk score for all HIV-1 serodiscordant couples; couples with a risk score ≥5 met the study eligibility criteria. Couples who had a HIV-1 risk score below the “high risk” cutoff (<5) were counseled about the prevalence and significance of HIV-1 serodiscordant results, behavioral risk reduction, the importance of condom use to reduce the risk of HIV-1 transmission, and their transmission risk. They were also referred to local public health clinics for ART provision according to national guidelines, male circumcision, and condoms. For couples who had a risk score ≥5, additional eligibility requirements included having adequate renal function and not having hepatitis B infection for HIV-1 uninfected partners. We excluded couples in whom the HIV-1 infected partner was already using of ART or had any WHO stage III or IV condition so that the research process would not distract from the urgent need for these individuals to initiate ART. HIV-1 uninfected women were excluded if they were pregnant or breastfeeding. Eligibility criteria were reviewed in totality to determine which couples met all criteria and were offered enrollment.

### Statistical analysis

To understand the ability of the HIV-1 risk score to identify high risk couples for antiretroviral-based prevention in the setting of an implementation project, we used descriptive statistics to summarize the proportion of screened couples that had an HIV-1 risk score ≥5 and the proportion of higher-risk couples that enrolled in the Partners Demonstration Project. Analyses were done using STATA version 13.1 (StataCorp, College Station, TX).

## Results

We screened and scored 1694 HIV-1 serodiscordant couples for eligibility to the Partners Demonstration Project and enrolled 1013 (59.8 %) couples giving a screen to enroll ratio of 1.7:1.

### Feasibility of using the HIV-1 risk score to identify high risk couples

**Fig. 2:**
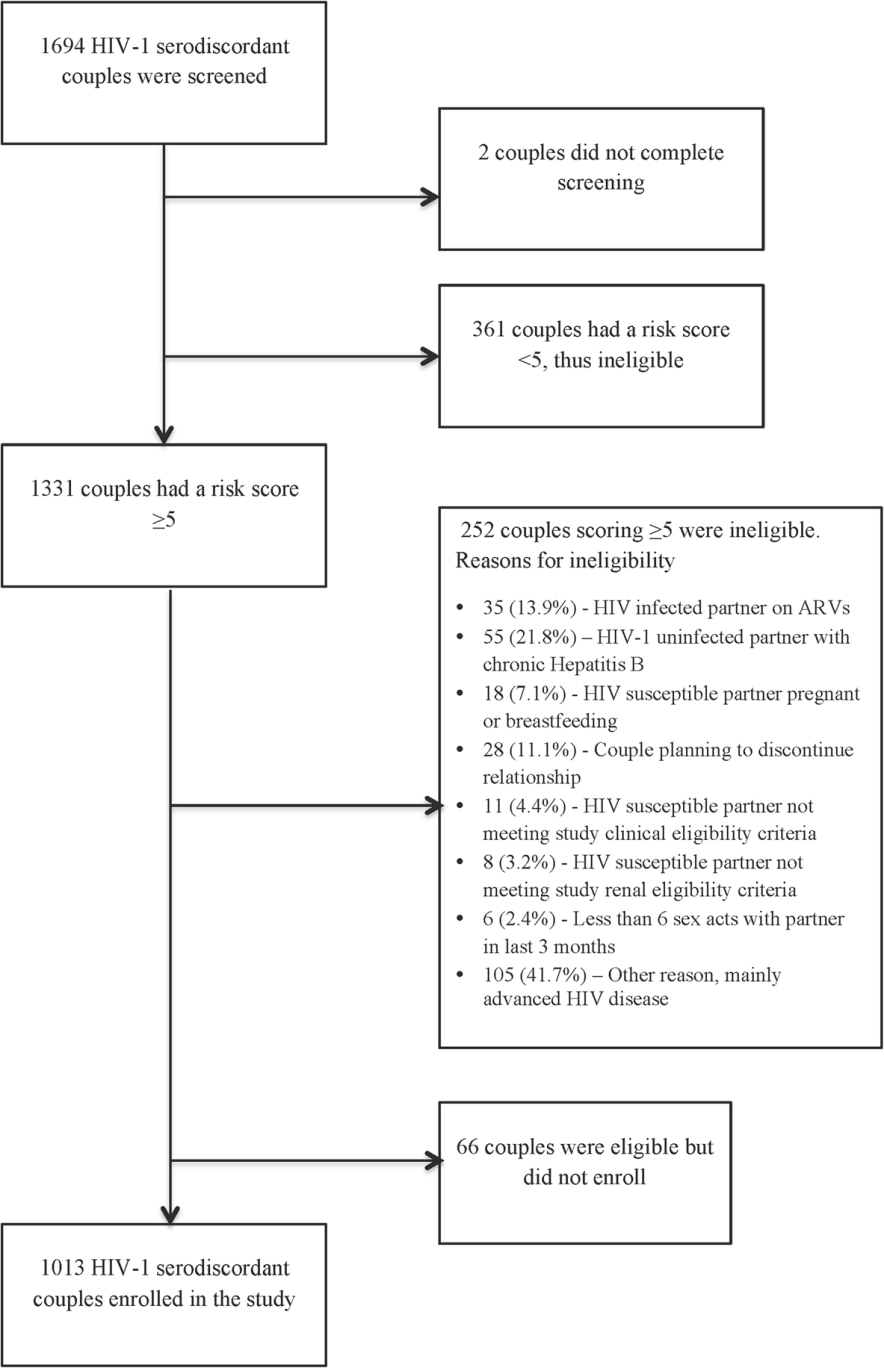
Flow chart of HIV-1 serodiscordant couples screened for eligibility to the Partners Demonstration Project

Among screened couples, 43 (2.5 %) had an HIV-1 risk score of 0–2, 319 (18.8 %) scored 3–4, and 1331 (78.6 %) scored ≥5 (the higher-risk population, Fig. 2). One couple that did not complete screening was not scored. There were 681 HIV-1 serodiscordant couples that were screened for the study but did not enroll: 613 (90.0 %) were not eligible, 66 (9.7 %) were eligible but did not enroll and 2 (0.3 %) did not complete screening. Of the ineligible HIV-1 serodiscordant couples, 252 (41.1 %) had a HIV-1 risk score ≥5 and the main reasons for their ineligibility were advanced clinical HIV-1 disease (41.7 %), infection with hepatitis B (21.8 %) or use of ART (13.9 %). Three-quarters (76 %) of HIV-1 serodiscordant couples scoring ≥5 enrolled into the study. Of the enrolled couples, 479 (47.3 %) scored >7, a level of risk with HIV-1 incidence >7 % per year in prior cohorts [10].

### Demographic characteristics of enrolled couples

**Fig. 3:**
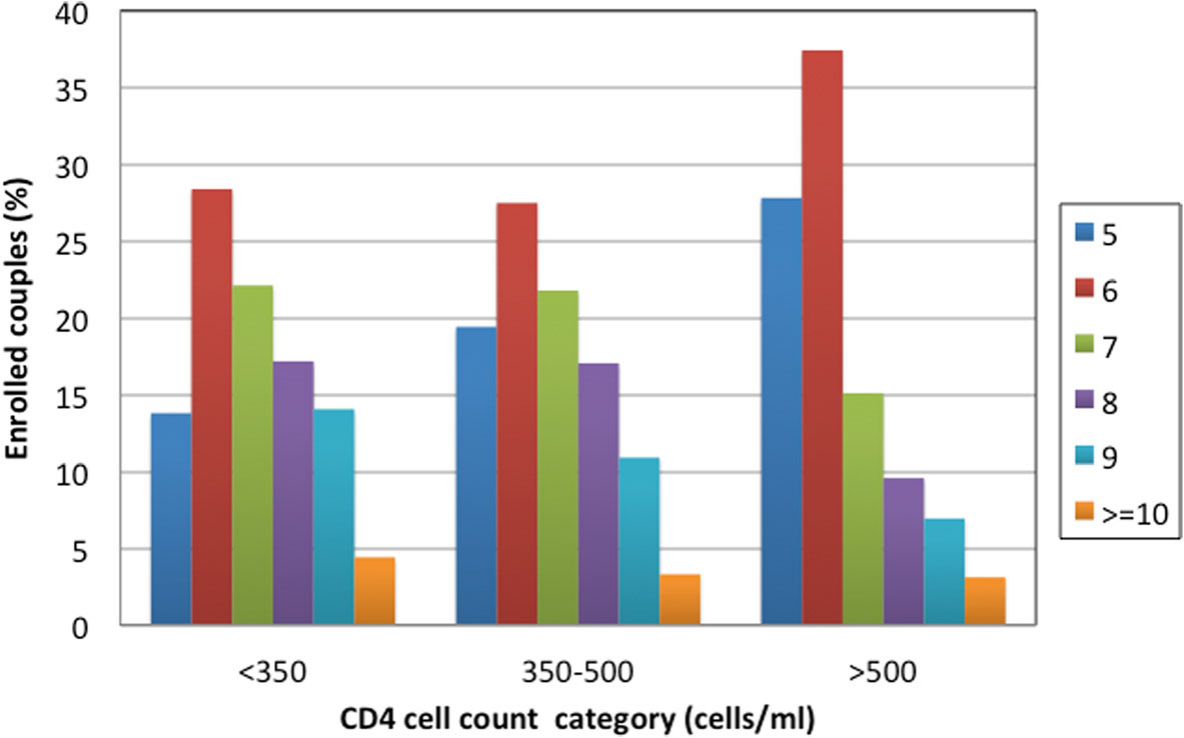
Proportion of enrolled couples in each CD4 category, by risk score

The median age of the HIV-1 uninfected partner was 29 years [interquartile range (IQR) 26–36], and 20 % were <25 years of age (Table 1). The HIV-1 uninfected partner was male in 67 % of the partnerships. Most couples (97.8 %) were married or cohabitating and had been living together for a median of 2.5 years [IQR 0.8–7.0] but had only learned of their HIV-1 serodiscordant status a median of 1 month [IQR 1–3] prior to the screening date. Over half of the couples (56.5 %) had no children and 64.8 % reported sex unprotected by condoms in the month prior to enrollment. A third of HIV-1 uninfected males were not circumcised. The median age of HIV-1 infected partners was 28 years [IQR 23–35] and 41.8 % had plasma HIV-1 RNA levels ≥50,000 copies/mL (median 4.6 log_10_ copies/mL, IQR 3.8–5.0). The median CD4 count was 436 cells/uL [IQR 272–638] and 41 % had a CD4 count >500 cells/μL. More than a third (34.7 %) of HIV-1 infected partners with CD4 counts >500 cells/μL were in partnerships that had a HIV-1 risk score ≥7 (Fig. 3). For twelve of the enrolled participants baseline plasma HIV-1 RNA levels were detectable even though the HIV-1 antibody test was negative, suggesting early HIV infection. Eligible couples who did not enroll had similar characteristics to enrolled couples.

**Table 1:** Baseline characteristics of enrolled HIV-1 serodiscordant couples in the Partners Demonstration Project (*N =* 1013)

	Median [IQR] or N (%)
Age of HIV-1 uninfected partner	29 (26–36)
18–20	38 (3.8)
21–30	534 (52.7)
≥ 31	441 (43.5)
Number of children within partnership^a^	0 (0–1)
0	572 (56.5)
1–2	334 (33.0)
≥ 3	107 (10.6)
Not circumcised (male HIV-1 uninfected only)	225 (33.1)
Married/ cohabitating with study partner^a^	991 (97.8)
Unprotected sex in month prior to enrollment^a^	656 (64.8)
Viral load of HIV-1 infected partner	4.6 (3.8–5.0)
< 10,000	280 (28.1)
10,000–49,999	301 (30.2)
≥ 50,000	417 (41.8)

^a^Data obtained from HIV-1 uninfected partners

## Discussion

In the Partners Demonstration Project, the use of a validated HIV-1 risk score identified heterosexual HIV-1 serodiscordant couples with characteristics consistent with high-risk of HIV-1 transmission. The score is comprised of easy to measure behavioral and biologic risk factors for HIV-1 [10, 12] and those with high scores are a natural priority population for coordinated delivery of early ART and time-limited PrEP prior to ART initiation and viral suppression. Three-quarters of the couples with a high score (≥5) were motivated to join the study and initiate PrEP and/or ART for HIV-1 prevention.

The risk score is a composite variable that facilitates the assessment of the distribution of risk within a HIV-1 serodiscordant partnership. A couple may have high risk on some factors and low on others, but the risk score tool enables computation of a level of overall risk for the partnership. Of note, we used a scoring tool that incorporates HIV-1 plasma viral load but a tool without viral load was also validated for use in places where such testing is not routinely available [10, 12]. Upon exclusion of viral load from the scoring tool a cut off of ≥4 would distinguish couples with an expected incidence >3 % per year and if applied to this cohort, 92 % of the couples would remain eligible and 66 % of the ineligible couples would still screen out.

In our prior randomized clinical trial of PrEP efficacy and safety (the Partners PrEP Study), eligibility criteria were similar minus the use of a risk scoring tool, study sites included the 4 that were part of the current study, and similar strategies were employed to recruit HIV serodiscordant couples [5]. Qualitatively, participants recruited into the Partners Demonstration Project were younger (median age 29 years in the Partners Demonstration Project versus 33 years in the Partners PrEP Study), had fewer children (median of 0 in the Partners Demonstration Project versus 2 in the Partners PrEP Study), had a higher proportion of unreported sex prior to enrollment (65 % in Partners Demonstration Project versus 27 % in the Partners PrEP Study), and had higher plasma HIV-1 RNA concentrations (4.6 log copies/ml in Partners Demonstration Project versus 3.9 log copies/ml in Partners PrEP Study). These characteristics all indicate higher HIV-1 risk in the cohort that was recruited using the scoring tool.

The risk score identifies HIV-1 serodiscordant couples with high risk of transmission for whom extra effort should be taken to overcome barriers to using HIV-1 prevention methods, including delays in ART initiation. Many HIV-1 infected partners in this study had high CD4 counts and by current guidelines, their indication for ART initiation was for the prevention benefit of their uninfected partner [12, 13]. Counseling of HIV-1 serodiscordant couples should emphasize the clinical and prevention benefits of ART, including for HIV-1 infected persons with higher CD4 and who are asymptomatic. In one review of ART initiation among HIV-1 infected partners in HIV-1 serodiscordant partnerships in East Africa, half of those found to be eligible delayed ART initiation by more than six months, with the delay more pronounced for those with higher CD4 counts [14]. Importantly, for maximal public health impact, PrEP may be reserved for couples during periods when they are at high risk of HIV-1 transmission. Thus, for the Partners Demonstration Project, where the goal was to demonstrate that higher-risk couples could be recruited and retained, couples who did not meet the risk score criteria were counseled about their ongoing HIV-1 risk and referred to public health clinics where they could access ART, as well as counseling services, STI screening and treatment, and medical male circumcision. PrEP is an effective strategy for HIV-1 serodiscordant couples when the infected partner delays ART initiation or for a time-limited period (e.g., 6 months) after ART initiation by the infected partner prior to becoming virally suppressed [15]. Future PrEP programs may use a similar scoring criterion to prioritize subsets of couples for PrEP; local context, including resource availability, would likely also shape prioritization decisions.

One limitation of our findings is that the majority of couples were recruited from voluntary counseling and testing centers, demonstrating motivation and health-seeking behavior, thus potentially impacting the generalizability to settings with provider-initiated counseling and testing and other testing strategies [16, 17]. Furthermore, we assessed feasibility of using the HIV-1 risk score within the Partners Demonstration Project at research clinics. Thus, the use of this score within a public health setting has not been evaluated. However, the Partners Demonstration Project was designed to reflect operations of public health clinics in Kenya and Uganda as much as possible.

Antiretroviral-based HIV-1 prevention interventions will achieve the greatest impact among HIV-1 serodiscordant couples if they are implemented with strategic and cost-effective provision to motivated couples at highest risk of HIV-1 transmission [9]. The HIV-1 risk score, an easy-to-use tool that utilizes information routinely collected in HIV prevention counseling sessions, identified this high-risk sub-population and prioritized them for antiretroviral based HIV-1 prevention interventions. The risk score is a pragmatic and inexpensive method that public health clinics could employ to help identify couples that would benefit most from an antiretroviral-based HIV-1 prevention intervention.

## Abbreviations

ART: Antiretroviral therapy
HBsAg: Hepatitis B surface antigen
IQR: Interquartile range
PMTCT: Prevention of mother-to-child HIV-1 transmission
PrEP: Pre-exposure prophylaxis
VCT: Voluntary counseling and testing

## References

[1] Dunkle KL, Stephenson R, Karita E, Chomba E, Kayitenkore K, Vwalika C, Greenberg L, Allen S (2008). New heterosexually transmitted HIV infections in married or cohabiting couples in urban Zambia and Rwanda: an analysis of survey and clinical data. Lancet.

[2] De Walque D (2007). Sero-discordant couples in five African countries: Implications for prevention strategies. Popul Dev Rev.

[3] Gray R, Ssempiija V, Shelton J, Serwadda D, Nalugoda F, Kagaayi J, Kigozi G, Wawer MJ (2011). The contribution of HIV-discordant relationships to new HIV infections in Rakai, Uganda. Aids.

[4] Cohen MS, Chen YQ, McCauley M, Gamble T, Hosseinipour MC, Kumarasamy N, Hakim JG, Kumwenda J, Grinsztejn B, Pilotto JH (2011). Prevention of HIV-1 infection with early antiretroviral therapy. N Engl J Med.

[5] Baeten JM, Donnell D, Ndase P, Mugo NR, Campbell JD, Wangisi J, Tappero JW, Bukusi EA, Cohen CR, Katabira E (2012). Antiretroviral prophylaxis for HIV prevention in heterosexual men and women. N Engl J Med.

[6] El-Sadr WM, Coburn BJ, Blower S (2011). Modeling the impact on the HIV epidemic of treating discordant couples with antiretrovirals to prevent transmission. AIDS.

[7] Hallett TB, Baeten JM, Heffron R, Barnabas R, de Bruyn G, Cremin I, Delany S, Garnett GP, Gray G, Johnson L (2011). Optimal uses of antiretrovirals for prevention in HIV-1 serodiscordant heterosexual couples in South Africa: a modelling study. PLoS Med.

[8] Curran K, Baeten JM, Coates TJ, Kurth A, Mugo NR, Celum C (2012). HIV-1 prevention for HIV-1 serodiscordant couples. Curr HIV/AIDS Rep.

[9] Cremin I, Hallett T (2015). Estimating the range of potential epidemiological impact of pre-exposure prophylaxis: run-away success or run-away failure?. AIDS (London, England).

[10] Kahle EM, Hughes JP, Lingappa JR, John-Stewart G, Celum C, Nakku-Joloba E, Njuguna S, Mugo N, Bukusi E, Manongi R (2013). An empiric risk scoring tool for identifying high-risk heterosexual HIV-1-serodiscordant couples for targeted HIV-1 prevention. J Acquir Immune Defic Syndr.

[11] Baeten J, Heffron R, Kidoguchi L, et al. Near elimination of HIV transmission in a demonstration project of PrEP and ART [abstract 24]. Presented at: CROI 2015. Seattle: Conference on Retroviruses and Opportunistic Infections. 2015.

[12] Ministry of Health. Guidelines on Use of Antiretroviral Drugs for Treating and Preventing HIV Infection: A rapid advice. Nairobi: National AIDS and STI Control Program (NASCOP); 2014.

[13] WHO. Guidance on Couples HIV Testing and Counselling Including Antiretroviral Therapy for Treatment and Prevention in Serodiscordant Couples. Geneva: World Health Organisation; 2012. 23700649

[14] Mujugira A, Celum C, Thomas KK, Farquhar C, Mugo N, Katabira E, Bukusi EA, Tumwesigye E, Baeten JM (2014). Delay of antiretroviral therapy initiation is common in East African HIV-infected individuals in serodiscordant partnerships. J Acquir Immune Defic Syndr.

[15] Mujugira A, Thomas K, Celum C, Farquhar C, Baeten JM, D D, Bukusi E. HIV-1 Transmission Risk Persists During the First 6 Months of Antiretroviral Therapy [Abstract 989]. Presented at: CROI 2015. Seattle: Conference on Retroviruses and Opportunistic Infections; 2015.

[16] Barnabas RV, van Rooyen H, Tumwesigye E, Murnane PM, Baeten JM, Humphries H, Turyamureeba B, Joseph P, Krows M, Hughes JP (2014). Initiation of antiretroviral therapy and viral suppression after home HIV testing and counselling in KwaZulu-Natal, South Africa, and Mbarara district, Uganda: a prospective, observational intervention study. The Lancet HIV.

[17] Genberg BL, Naanyu V, Wachira J, Hogan JW, Sang E, Nyambura M, Odawa M, Duefield C, Ndege S, Braitstein P (2015). Linkage to and engagement in HIV care in western Kenya: An observational study using population-based estimates from home-based counseling and testing. The lancet HIV.

